# Sequence analysis of four vitamin D family genes (*VDR, CYP24A1, CYP27B1* and *CYP2R1*) in Vogt-Koyanagi-Harada (VKH) patients: identification of a potentially pathogenic variant in *CYP2R1*

**DOI:** 10.1186/s12886-016-0354-6

**Published:** 2016-10-04

**Authors:** Ma’an Abdullah Al-Barry, Alia M Albalawi, Mohammed Abu Sayf, Abdulrahman Badawi, Sibtain Afzal, Muhammad Latif, Mohammed I. Samman, Sulman Basit

**Affiliations:** 1 College of Medicine, Taibah University Almadinah Almunawarah, Medina, Kingdom of Saudi Arabia; 2 Magribi Hospital, Almadinah Almunawarah, Medina, Kingdom of Saudi Arabia; 3 Center for Genetics and Inherited Diseases, Taibah University Almadinah Almunawarah, Medina, 30001 Kingdom of Saudi Arabia; 4 Prince Naif Center for Immunology Research, College of Medicine, King Saud University, Riyadh, 11472 Saudi Arabia

**Keywords:** Vogt-Koyanagi-Harada, Vitamin D, Genes, Mutations

## Abstract

**Background:**

VKH is a rare autoimmune disease. Decreased level of vitamin D has recently been found to be involved in the pathogenesis of Vogt-Koyanagi-Harada (VKH) disease. This study was designed to screen the vitamin D pathway genes for pathogenic mutations, if any, in VKH patients.

**Methods:**

Genomic DNA was extracted from blood samples collected from patients with VKH disease and healthy controls. Entire coding region, exon-intron junctions of four genes were sequenced in DNA from 39 Saudi VKH patients and 50 ethnically matched healthy individuals. All patients and controls were unrelated.

**Results:**

Vitamin D levels in VKH patients were found either insufficient (21–29 ng/mL) or deficient (<20 ng/mL). Sequencing analysis of the *VDR, CYP24A1, CYP27B1* and *CYP2R1* detected twelve nucleotide changes in these genes in our cohort of 39 patients; 4 of which were non-coding, 6 were synonymous coding and 2 were non-synonymous coding sequence changes. All synonymous coding variants were benign polymorphisms with no apparent clinical significance. A non-synonymous coding sequence variant (c.2 T > C; p.1Met?) found in *VDR* is an initiation coding change and was detected in control individuals as well, while another variant (c.852G > A; p.284 M > I) found in *CYP2R1* is predicted to be disease causing by mutationtaster software. This potentially pathogenic variant was found in 17 out of 39 VKH patients.

**Conclusions:**

Screening of four Vitamin D pathway genes in 39 VKH patients shows that a potentially pathogenic sequence variant in CYP2R1 may cause VKH in a subset of patients. These findings support the previous observation that low vitamin D levels might play a role in VKH pathogenesis and mutations in genes involved in vitamin D anabolism and catabolism might be of importance in VKH pathobiology.

## Background

Vogt-Koyanagi-Harada (VKH) syndrome is a rare multisystem autoimmune disease that affects melanin containing tissues, including the eye, inner ear, meninges and skin. The disease is characterized by bilateral uveitis associated with a varying degree of auditory, neurological and cutaneous manifestations [1–3]. VKH affects more frequently people with darker skin pigmentation. Asians, Native Americans, Middle Easterners and Hispanics are most frequently affected [4]. It predominates in patients aged between 20 and 50 years with a female:male ratio of 2:1.

The classic clinical course is characterized by severe bilateral granulomatous panuveitis, hypoacusis and meningitis in addition to cutaneous involvement with poliosis, vitiligo and alopecia. If not treated appropriately it results in severely decreased vision or even leads to blindness [2, 5, 6]. Although the exact cause of VKH disease remains unclear, studies have shown that an autoimmune response directed against melanocytes plays a major role in the onset of this disease [7, 8]. Although a number of HLA and non-HLA genes have been shown to be associated with VKH [9–12] the genetic basis of VKH still remain illusive. Therefore, further studies on the association of autoimmune modulatory genes may yield informative data for the genetic background of VKH disease.

Vitamin D is produced in the skin or obtained from the diet [13]. Its receptor has been found in the immune cells, and some immune cells are able to produce Vitamin D3 [14–17]. The biologically active metabolite of Vitamin D3, 1,25(OH)_2_D_3_, has been shown to have immunomodulatory action alongside its role in the bone and calcium metabolism [18, 19]. Vitamin D receptor (VDR) gene negative mice showed a significantly increased susceptibility to several autoimmune diseases, such as autoimmune encephalomyelitis [20, 21], autoimmune uveitis [22] and allergic asthma [23]. Moreover, multiple studies found decreased levels of serum Vitamin D in several human autoimmune diseases, such as multiple sclerosis [24–26], rheumatoid arthritis [27, 28], Behçet’s disease [29], Graves disease [30] and systemic lupus erythematosus [31]. It has been reported that decreased 1,25(OH)_2_D_3_ level may play a role in the development of VKH disease [32]. Vitamin D deficiency, compromising the immunoregulatory action leading to the autoimmune diseases like VKH could result from either environmental factors or defect in genes concern with Vitamin D metabolism pathway or both. We, therefore, entertained the hypothesis that genetic variation in the Vitamin D genes could be associated with VKH disease.

In Saudi Arabia, VKH is a common cause of uveitis [33] but no study has been conducted to explore the role of vitamin D pathway genes in VKH pathogenesis. In the present study, we screened Vitamin D metabolism pathway genes (*VDR, CYP24A1, CYP27B1 and CYP2R1*) to examine the possible involvement of variation in these genes with VKH disease in Saudi patients. We identified a novel missense variant in *CYP2R1* in VKH patients which might be responsible for low vitamin D level in these patients. Overall, our results showed that a variation/polymorphisms in Vitamin D pathway genes tested here are not responsible for VKH in Saudi population. However, we detected a variant in *CYP2R1* gene that may be pathogenic for VKH disease.

## Methods

### Subjects

All subjects were recruited from Magrabi Hospital Almadinah Almunawarah. We collected 39 VKH patients and 50 control individuals for this study. All affected and control individuals signed informed written consent prior to start of the study. In case of minor, consent was taken from parents. All patients were examined clinically by a senior ophthalmologist and diagnosed as VKH. Revised diagnostic criteria has been used for VKH diagnosis [3]. Systemic observations for vertigo, poliosis and alopecia, vitiligo, hearing impairment and tinnitus were recorded for all VKH patient. Ethical approval for the study was obtained from the IRB of the Center for Genetics and Inherited Diseases (CGID), Taibah University Almadinah Almunawarah. All experimental procedures were conducted in accordance with the tenets of the Declaration of Helsinki.

All patients were diagnosed using slit lamp bio-microscopy while cornea was found clear. Fundus examination was carried out using indirect ophthalmoscope and a 20× diopter aspheric lens.

### Blood collection and gDNA extraction

In this study, we screened 39 VKH patients and 50 controls for 4 vitamin D pathway genes. Peripheral blood samples of 6 ml was collected from each of the patients and the controls in EDTA tubes. Extraction of genomic DNA was performed using Qiagen blood mini kit. DNA was quantified using Maestro spectrophotometer.

### Vitamin D measurement

Serum levels of 25-hydroxyvitamin D3 (25OHD3) were measured in all 39 VKH patients and 50 controls by radioimmunoassay using the Wallac 1470 Gamma Counter (Wallac Inc, Gaithersburg, MD, USA). 25OHD3 level of >30 ng/mL was considered normal. Vitamin D deficiency was defined as a serum level of 25OHD of ≤20 ng/mL and insufficiency as a serum level between 21–29 ng/mL.

### Sequencing of vitamin D pathway genes

gDNA was diluted to 20 ng/ul concentration and PCR amplification of coding regions of all four genes was performed using primers flanking exons following a protocol used earlier [34]. Primer sequences are available on request. Bidirectional sequencing of all fragments was carried out using BigDye (Applied Biosystems, Foster city, CA) chain termination chemistry. Fragments were then separated on AB 3500 genetic analyzer (Life Technologies). All sequenced fragments were analyzed using BioEdit software (http://www.mbio.ncsu.edu/bioedit/bioedit.html) and compared to the reference sequences of corresponding genes from UCSC genome browser (http://genome.ucsc.edu/cgi-bin/hgGateway).

In house controls were used and it was ensured that controls are healthy individuals without having any ocular disease(s) or previous ophthalmic surgeries.

## Results

### Clinical description of subjects

In this study, 39 unrelated VKH patients (Table 1) and 50 unrelated controls were screened for four genes by Sanger sequencing. Of the 39 VKH patients there were 27 females and 12 males with a mean age of 32.58 years. Of the 50 controls there were 29 females and 21 males with a mean age of 34.75.

**Table 1:** Clinical characteristics and genetic variants identified in VKH patients

Patient ID	Age/Sex	Age at onset	Vitamin D level	Clinical description	Variant identified
VKH1	40–45/M	40–45 (A)	14.4 ng/mL	De-pigmented fundus OU, Keratoconus OU, Diffuse vitiligo, Poor improvement of VA on treatment	CYP24A1 (g. 632 T > G; c.234 T > G) VDR (g.63937 T > C; p.1Met?, g.65058 T > C; c.1056 T > C)
VKH2	16–20/M	16–20 (C)	16 ng/mL	De-pigmented fundus OU, Vertigo, Tinnitus, Improved VA on treatment	VDR (g.63937 T > C; p.1Met?) CYP2R1 (c.852G > A; p.284 M > I)
VKH3	10–15/F	6–10 (A)	23 ng/mL	De-pigmented fundus OU, Band keratopathy, Vertigo, Tinnitus, Maintained VA, Vitiligo, Poliosis	CYP27B1 (g.2989C > T) VDR (g.63937 T > C; p.1Met?, g.64978 G > T; -49 int 9G > T)
VKH4	16–20/M	17 (A)	13 ng/mL	De-pigmented fundus OU, CNVM, Vertigo, Tinnitus, Decrease hearing, Improved VA on treatment	CYP27B1 (g.2989C > T) VDR (g.64978 G > T; -49 int 9G > T)
VKH5	30–35/M	30–35 (A)	17 ng/mL	De-pigmented fundus OU, Improved VA on treatment	CYP24A1 (g.512G > T; c.114G > T, g. 632 T > G; c.234 T > G, g.821C > T)
VKH6	46–50/F	36–40 (A)	14 ng/mL	De-pigmented fundus OU, Peripheral Anterior Synechea, Improved VA on treatment, Vitiligo	CYP2R1 (c.852G > A; p.284 M > I) CYP24A1 (g.821C > T)
VKH7	20–25/F	20–25 (A)	22 ng/mL	De-pigmented fundus OU, Cataract, Improved VA on treatment	VDR (g.63937 T > C; p.1Met?)
VKH8	56–60/F	56–60 (C)	26 ng/mL	De-pigmented fundus OU, Improved VA on treatment	CYP2R1 (c.852G > A; p.284 M > I)
VKH9	30–35/F	30–35 (A)	16 ng/mL	De-pigmented fundus OU, Improved VA on treatment, Alopecia, Vitiligo	CYP24A1 (g.512G > T; c.114G > T)
VKH10	50–55/M	50–55 (A)	11 ng/mL	De-pigmented fundus OU, Cataract, Improved VA on treatment	CYP24A1 (g. 632 T > G; c.234 T > G)
VKH11	16–20/F	16–20 (A)	15 ng/mL	De-pigmented fundus OU, Improved VA on treatment	CYP2R1 (c.852G > A; p.284 M > I) VDR (g.63937 T > C; p.1Met?)
VKH12	36–40/M	36–40 (Acute)	24 ng/mL	De-pigmented fundus OU, Improved VA on treatment	CYP27B1 (g.2989C > T)
VKH13	56–60/F	56–60 (A)	22 ng/mL	De-pigmented fundus OU, Improved VA on treatment	CYP24A1 (g.512G > T; c.114G > T, g.2989C > T)
VKH14	40–45/F	30–35 (A)	25 ng/mL	De-pigmented fundus OU, Improved VA on treatment, Alopecia, Vitiligo	VDR (g.65058 T > C; c.1056 T > C)
VKH15	16–20/F	10–15 (A)	13 ng/mL	De-pigmented fundus OU, Improved VA on treatment, Alopecia, Vitiligo, Poliosis	VDR (g.63937 T > C; p.1Met?)
VKH16	30–35/F	10–15 (A)	12 ng/mL	De-pigmented fundus OU, Improved VA on treatment, Vitiligo	CYP24A1 (g.2989C > T)
VKH17	20–25/M	16–20 (A)	17 ng/mL	De-pigmented fundus OU, Peripheral Anterior Synechea, Improved VA on treatment, Vitiligo	VDR (g.64978 G > T;–49 int 9G > T) CYP24A1 (g.2989C > T)
VKH18	30–35/M	30–35 (A)	25 ng/mL	De-pigmented fundus OU, Keratoconus OU, Diffuse vitiligo, Poor improvement of VA on treatment	CYP2R1 (c.852G > A; p.284 M > I) CYP24A1 (g.2989C > T)
VKH19	40–46/M	36–40 (A)	17 ng/mL	De-pigmented fundus OU, Band keratopathy, Vertigo, Tinnitus, Maintained VA, Vitiligo, Poliosis	CYP2R1 (c.852G > A; p.284 M > I) CYP24A1 (g.512G > T; c.114G > T, g.2989C > T)
VKH20	20–25/F	20–25 (A)	13 ng/mL	De-pigmented fundus OU, Cataract, Improved VA on treatment	VDR (g.64978 G > T;–49 int 9G > T)
VKH21	50–55/M	46–50 (C)	26 ng/mL	De-pigmented fundus OU, Peripheral Anterior Synechea	CYP24A1 (g.2989C > T)
VKH22	30–35/F	30–35 (A)	12 ng/mL	De-pigmented fundus OU, Improved VA on treatment, Alopecia, Vitiligo	CYP2R1 (c.852G > A; p.284 M > I) VDR (g.64978 G > T;–49 int 9G > T)
VKH23	16–20/F	16–20 (A)	17 ng/mL	De-pigmented fundus OU, Cataract, Improved VA on treatment	CYP27B1 (g.2989C > T)
VKH24	30–35/F	30–35 (A)	23 ng/mL	De-pigmented fundus OU, Improved VA on treatment, Alopecia, Vitiligo	CYP2R1 (c.852G > A; p.284 M > I)
VKH25	46–50/F	36–40 (A)	27 ng/mL	De-pigmented fundus OU	CYP2R1 (c.852G > A; p.284 M > I)
VKH26	40–45/M	36–40 (C)	12 ng/mL	De-pigmented fundus OU, Peripheral Anterior Synechea, Improved VA on treatment, Vitiligo	VDR (g.63937 T > C; p.1Met?) CYP2R1 (c.852G > A; p.284 M > I)
VKH27	56–60/M	50–55 (C)	16 ng/mL	De-pigmented fundus OU	CYP27B1 (g.2989C > T)
VKH28	26–30/F	26–30 (A)	25 ng/mL	De-pigmented fundus OU, Cataract, Improved VA on treatment	CYP24A1 (g.512G > T; c.114G > T)
VKH29	40–45/F	40–45 (C)	14 ng/mL	De-pigmented fundus OU, Improved VA on treatment, Alopecia, Vitiligo	CYP27B1 (g.2989C > T)
VKH30	50–55/M	26–30 (A)	25 ng/mL	De-pigmented fundus OU, Keratoconus OU, Diffuse vitiligo, Poor improvement of VA on treatment	CYP2R1 (c.852G > A; p.284 M > I) VDR (g.63937 T > C; p.1Met?)
VKH31	26–30/F	20–25 (A)	15 ng/mL	De-pigmented fundus OU, Improved VA on treatment	CYP2R1 (c.852G > A; p.284 M > I) VDR (g.63937 T > C; p.1Met?)
VKH32	40–45/F	16–20 (A)	27 ng/mL	De-pigmented fundus OU	CYP2R1 (c.852G > A; p.284 M > I)
VKH33	40–45/M	36–40 (C)	12 ng/mL	De-pigmented fundus OU, Peripheral Anterior Synechea, Improved VA on treatment, Vitiligo	CYP2R1 (c.852G > A; p.284 M > I) VDR (g.63937 T > C; p.1Met?)
VKH34	50–55/M	50–55 (C)	16 ng/mL	De-pigmented fundus OU	CYP27B1 (g.2989C > T)
VKH35	26–30/F	20–25 (A)	25 ng/mL	De-pigmented fundus OU, Cataract, Improved VA on treatment	CYP24A1 (g.512G > T; c.114G > T)
VKH36	40–45/F	40–45 (C)	14 ng/mL	De-pigmented fundus OU, Improved VA on treatment, Alopecia, Vitiligo	CYP27B1 (g.2989C > T)
VKH37	26–30/F	20–25 (A)	15 ng/mL	De-pigmented fundus OU, Improved VA on treatment	CYP2R1 (c.852G > A; p.284 M > I) VDR (g.63937 T > C; p.1Met?)
VKH38	40–45/F	26–30 (A)	27 ng/mL	De-pigmented fundus OU	CYP2R1 (c.852G > A; p.284 M > I)
VKH39	46–50/F	26–30 (C)	26 ng/mL	De-pigmented fundus OU, Improved VA on treatment	CYP2R1 (c.852G > A; p.284 M > I)

*VKH* Vogt-Koyanagi-Harada, *M* Male, *F* Female, *A* Acute, *C* Chronic

History of ocular trauma and ocular surgery before the onset of the disease was ruled out in all patients. No sign of optic atrophy was found in all VKH patients. Distribution of eye involvement is bilateral affecting the whole middle layer of the eye (Pan-uveitis OU). All patients had depigmented fundus OU from mild to severe.

Best Characterized Visual Acuity (BCVA) in all VKH patients was observed in the range of 20/20–20/200 (BCVA). Patients with BCVA of 20/200 had severe depigmentation of the retina and significant retinal pigmentation of epithelium (RPE) changes in the macular area. Some patients developed complications including cataract, glaucoma, subretinal neovascular membranes and two patients developed subretinal fibrosis as well. Recurrence was observed in four cases.

All patients and controls were screened for serum vitamin D levels. Most of the patients were found Vitamin D deficient (Table 1). Among 50 controls, 12 were found Vitamin D deficient, 23 were Vitamin D insufficient and 15 showed Vitamin D levels of more than 30 ng/mL.

### Mutation detection

**Fig. 1:**
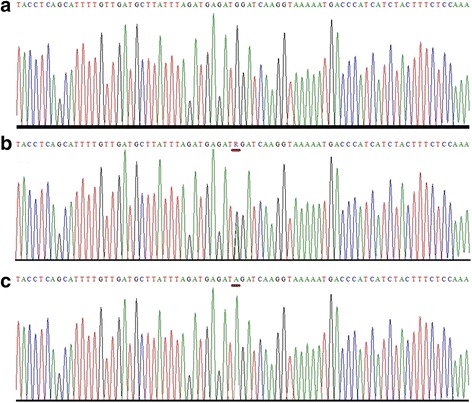
Sequence analysis of potentially pathogenic variant (c.852G > A) in *CYP2R1* gene. Partial DNA sequence of *CYP2R1* gene from (**a**) control individual, showing wild type sequence (**b**) a heterozygous carrier and (**c**) a homozygous (VKH patients) showing a transition (G > A). Mutated position is underlined

**Fig. 2:**
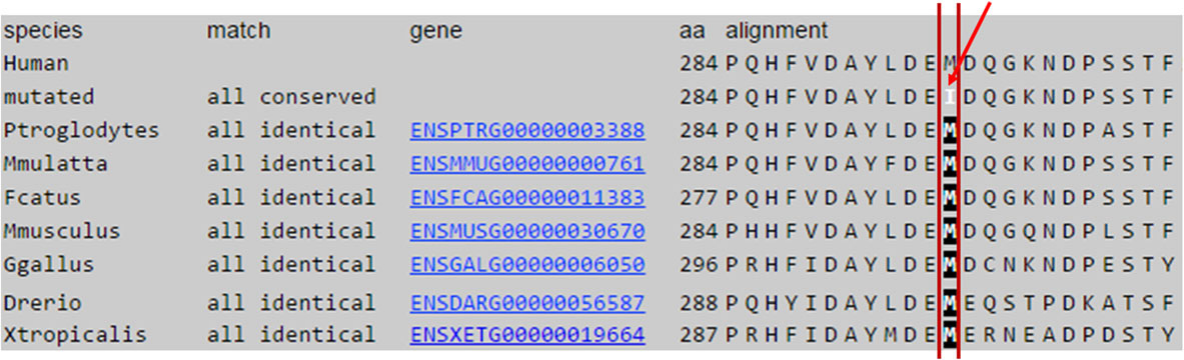
Comparison of partial amino acid sequence of human *CYP2R1* with other primates. The shaded Methionine (M) indicates the conserved residue across different species. Isoleucine (I) indicated by an arrow represent the mutated amino acid observed in the VKH patients in this study

The full coding region, exon-intron junctions and the 5′ and 3′UTRs of *VDR, CYP24A1, CYP27B1* and *CYP2R1* were sequenced in all 39 patients. Controls were screened only for the variants detected in patients. We detected twelve nucleotide changes in both patients and controls (Table 1). Of all these, 4 were non-coding (g.64978G > T in *VDR*, g.2989C > T in *CYP27B1*, g.821C > T and g.15916 T > C in *CYP24A1*), 6 were synonymous coding (c.1056 T > C; 352I > I in VDR, c.114G > T; p.37P > P, c.234 T > G; p.77G > G, c.469C > A; p. 156R > R, c.552C > T; p. 183A > A, c.1125G > A; p. 374P > P in CYP24A1) and 2 were non-synonymous coding sequence changes (c.2 T > C; p.1Met? in *VDR* and c.852G > A; p.284 M > I in *CYP2R1*). All non-coding and synonymous coding variants were benign polymorphisms with no apparent clinical significance. Non-synonymous coding sequence variant (c.2 T > C; p.1Met?) in *VDR* is an initiation coding change and was found in control individuals as well. Homozygous variant (c.852G > A; p.284 M > I) in *CYP2R1* was identified in 17 out of 29 patients and no control individual was found carrying the same variant. This variant (c.852G > A; p.284 M > I) in *CYP2R1* is predicted to be a disease causing by MutationTaster software (Fig. 1). Multiple sequence alignment shows that the amino acid methionine at position p.284 M is evolutionarily conserved (Fig. 2).

## Discussion

It is known that vitamin D plays an important role in melanin production and its deficiency has been shown to be associated with skin depigmentation [35]. In VKH patients, melanocytes tends to disappear from the outer layer of the choroid leading to depigmented Dellen-Fuchs scars. Moreover, inflammation of melanocytes of retinal pigment epithelium cause serous retinal detachment.

Recently polymorphisms in Vitamin D receptor (*VDR*) and 7-dehydrocholesterol reductase (*DHCR7*) genes have been associated with Behçet’s disease [36–38].

Moreover, studies examining *VDR* polymorphisms reported significant associations with diabetes, arthritis, autoimmune diseases and hypertension [39–42]. Significant association between polymorphisms in the *VDR* gene with asthma have also been reported in several genetic association studies [43, 44] but has not been consistently replicated [45]. Feng and colleagues showed significant association of autoimmune thyroid diseases (AITD) with *VDR* gene polymorphisms *TaqI* (rs731236) and *BsmI* (rs1544410) [46].

*VDR* encodes vitamin D receptor which shows high binding affinity for vitamin D3. Vitamin D3 binding activates VDR and ligand-activated VDR performs its function of gene expression by chromatin modification and the transcription regulation. DHCR7 coverts 7-dehydrocholesterol to cholesterol and, thus, reduces the substrate for vitamin D3 synthesis [47]. DHCR7 mutations have been shown to control vitamin D levels in serum [48, 49]. Similarly, low vitamin D serum level has been associated with VKH disease. Both Behçet’s disease and VKH manifest intraocular inflammation (Uveitis) which strengthens the hypothesis that variations in vitamin D pathway genes may cause VKH as well. The current study is based on this hypothesis and hence, we screened four vitamin D family genes (*VDR, CYP24A1, CYP27B1 and CYP2R1*) in 39 VKH patients to detect possible mutations underlying VKH in Saudi population. We identified various population polymorphisms in all these genes (Table 1). However, we identified a novel homozygous missense mutation (c.852G > A; p.284 M > I) in *CYP2R1* gene in 17 VKH patients (Fig. 1). This variant in not present in the homozygous state in 50 control individuals. This mutation changes a conserved amino acid methionine to Isoleucine. In silico analysis predicted that this mutation is probably pathogenic. I-Mutant software (used for prediction of protein stability upon single point mutation) predicted the mutant protein as less stable or with decreased stability [50]. Moreover, we used DUET (predicting effects of mutations on protein stability via an integrated computational approach), SDM (predicting effects of mutations on protein stability and malfunction) and mCSM (predicting the effect of mutation in protein using graph based signatures) for prediction of effect of mutation of the protein and found that this mutation is indeed destabilizing [51–53].

Failure to detect pathogenic variants in other vitamin D genes such as *CYP24A1*, *CYP27B1* and *VDR* does not rule out the possibility that other relevant vitamin D gene mutations could cause VKH in Saudi patients. Deep sequencing in a large number of samples would be required to find if any other Vitamin D pathway gene mutations are associated with VKH disease. Also, the non-genetic factors causing Vitamin D deficiency in these patients should be explored.

## Conclusions

These findings support the previous observation that low vitamin D levels might play a role in VKH pathogenesis and mutations in genes involved in vitamin D anabolism and catabolism might be of importance in VKH pathobiology. In conclusion, our study for the first time reports a potentially causative role of *CYP2R1* mutation in VKH disease. Studies on larger cohort of patients are needed to confirm this observation.

## References

[1] Norose K, Yano A (1996). Melanoma specific Th1 cytotoxic T lymphocyte lines in Vogt-Koyanagi-Harada disease. Br J Ophthalmol.

[2] Bykhovskaya I, Thorne JE, Kempen JH, Dunn JP, Jabs DA (2005). Vogt-Koyanagi-Harada disease: clinical outcomes. Am J Ophthalmol.

[3] Read RW (2002). Vogt-Koyanagi-Harada disease. Ophthalmol Clin North Am.

[4] Iqniebi A, Gaafar A, Sheereen A, Al-Suliman A, Mohamed G, Al-Hussein K, Tabbara KF (2009). HLA-DRB1 among patients with Vogt-Koyanagi-Harada disease in Saudi Arabia. Mol Vis.

[5] Yang P, Zhang Z, Zhou H, Li B, Huang X, Gao Y, Zhu L, Ren Y, Klooster J, Kijlstra A (2005). Clinical patterns and characteristics of uveitis in a tertiary center for uveitis in China. Curr Eye Res.

[6] Rao NA, Gupta A, Dustin L, Chee SP, Okada AA, Khairallah M, Bodaghi B, Lehoang P, Accorinti M, Mochizuki M, Prabriputaloong T, Read RW (2010). Frequency of distinguishing clinical features in Vogt-Koyanagi-Harada disease. Ophthalmology.

[7] Yamaki K, Gocho K, Hayakawa K, Kondo I, Sakuragi S (2000). Tyrosinase family proteins are antigens specific to Vogt-Koyanagi-Harada disease. J Immunol.

[8] Touitou V, Bodaghi B, Cassoux N, Tran TH, Rao NA, Cacoub P, LeHoang P (2005). Vogt-Koyanagi-Harada disease in patients with chronic hepatitis C. Am J Ophthalmol.

[9] Du L, Kijlstra A, Yang P (2009). Immune response genes in uveitis. Ocul Immunol Inflamm.

[10] Baba M, Imai T, Nishimura M, Kakizaki M, Takagi S, Hieshima K, Nomiyama H, Yoshie O (1997). Identification of CCR6, the specific receptor for a novel lymphocyte-directed CC chemokine LARC. J Biol Chem.

[11] Schutyser E, Struyf S, Van Damme J (2003). The CC chemokine CCL20 and its receptor CCR6. Cytokine Growth Factor Rev.

[12] Zamecki KJ, Jabs DA (2010). (2010) HLA typing in uveitis: use and misuse. Am J Ophthalmol.

[13] Basit S (2013). Vitamin D, in health and disease: a literature review. Br J Biomed Sci.

[14] van Etten E, Mathieu C (2005). Immunoregulation by 1,25-dihydroxyvitamin D3: basic concepts. J Steroid Biochem Mol Biol.

[15] Cantorna MT, Zhu Y, Froicu M, Wittke A (2004). Vitamin D status, 1,25-dihydroxyvitamin D3, and the immune system. Am J Clin Nutr.

[16] Nagpal S, Lu J, Boehm MF (2001). Vitamin D analogs: mechanism of action and therapeutic applications. Curr Med Chem.

[17] Tetlow LC, Smith SJ, Mawer EB, Woolley DE (1999). Vitamin D receptors in the rheumatoid lesion: expression by chondrocytes, macrophages, and synoviocytes. Ann Rheum Dis.

[18] Adams JS, Hewison M (2008). Unexpected actions of vitamin D: new perspectives on the regulation of innate and adaptive immunity. Nat Clin Pract Endocrinol Metab.

[19] Hewison M (2010). Vitamin D, and the immune system: new perspectives on an old theme. Endocrinol Metab Clin North Am.

[20] Nashold FE, Spach KM, Spanier JA, Hayes CE (2009). Estrogen controls vitamin D3-mediated resistance to experimental autoimmune encephalomyelitis by controlling vitamin D3 metabolism and receptor expression. J Immunol.

[21] Pedersen LB, Nashold FE, Spach KM, Hayes CE (2007). 1,25-dihydroxyvitamin D3 reverses experimental autoimmune encephalomyelitis by inhibiting chemokine synthesis and monocyte trafficking. J Neurosci Res.

[22] Tang J, Zhou R, Luger D, Zhu W, Silver PB, Grajewski RS, Su SB, Chan CC, Adorini L, Caspi RR (2009). Calcitriol suppresses antiretinal autoimmunity through inhibitory effects on the Th17 effector response. J Immunol.

[23] Wittke A, Weaver V, Mahon BD, August A, Cantorna MT (2004). Vitamin D receptor-deficient mice fail to develop experimental allergic asthma. J Immunol.

[24] Niino M (2010). Vitamin D, and its immunoregulatory role in multiple sclerosis. Drugs Today (Barc).

[25] Soilu-Hänninen M, Laaksonen M, Laitinen I, Eralinna JP, Lilius EM, Mononen I (2008). A longitudinal study of serum 25- hydroxyvitamin D and intact parathyroid hormone levels indicate the importance of vitamin D and calcium homeostasis regulation in multiple sclerosis. J Neurol Neurosurg Psychiatry.

[26] Barnes MS, Bonham MP, Robson PJ, Strain JJ, Lowe-Strong AS, Eaton-Evans J, Ginty F, Wallace JM (2007). Assessment of 25-hydroxyvitamin D and 1,25-dihydroxyvitamin D3 concentrations in male and female multiple sclerosis patients and control volunteers. Mult Scler.

[27] Cutolo M, Otsa K, Uprus M, Paolino S, Seriolo B (2007). Vitamin D in rheumatoid arthritis. Autoimmun Rev.

[28] Colin EM, Asmawidjaja PS, van Hamburg JP, Mus AM, van Driel M, Hazes JM, van Leeuwen JP, Lubberts E (2010). 1,25-dihydroxyvitamin D3 modulates Th17 polarization and interleukin-22 expression by memory T cells from patients with early rheumatoid arthritis. Arthritis Rheum.

[29] Do JE, Kwon SY, Park S, Lee ES (2008). Effects of vitamin D on expression of Toll-like receptors of monocytes from patients with Behcet’s disease. Rheumatology (Oxford).

[30] Misharin A, Hewison M, Chen CR, Lagishetty V, Aliesky HA, Mizutori Y, Rapoport B, McLachlan SM (2009). Vitamin D deficiency modulates Graves’ hyperthyroidism induced in BALB/c mice by thyrotropin receptor immunization. Endocrinology.

[31] Borba VZ, Vieira JG, Kasamatsu T, Radominski SC, Sato EI, Lazaretti-Castro M (2009). Vitamin D deficiency in patients with active systemic lupus erythematosus. Osteoporos Int.

[32] Yi X, Yang P, Sun M, Yang Y, Li F (2011). Decreased 1,25-Dihydroxyvitamin D3 level is involved in the pathogenesis of Vogt-Koyanagi-Harada (VKH) disease. Mol Vis.

[33] Tabbara KF, Chavis PS, Freeman WR (1998). Vogt-Koyanagi-Harada syndrome in children compared to adults. Acta Ophthalmol Scand.

[34] Basit S, Ali G, Wasif N, Ansar M, Ahmad W (2010). Genetic mapping of a novel hypotrichosis locus to chromosome 7p21.3-p22.3 in a Pakistani family and screening of the candidate genes. Hum Genet.

[35] AlGhamdi K, Kumar A, Moussa N (2013). The role of vitamin D in melanogenesis with an emphasis on vitiligo. Indian J Dermatol Venereol Leprol.

[36] Tizaoui K, Kaabachi W, Ouled Salah M, Ben Amor A, Hamzaoui A, Hamzaoui K (2014). Vitamin D receptor TaqI and ApaI polymorphisms: a comparative study in patients with Behçet’s disease and Rheumatoid arthritis in Tunisian population. Cell Immunol.

[37] Feng HM, Kuo SC, Chen CY, Yeh YW (2014). Nocturnal bruxism in a patient with Behçet disease and posttraumatic stress disorder successfully treated with gabapentin. Clin Neuropharmacol.

[38] Fang J, Hou S, Xiang Q, Qi J, Yu H, Shi Y, Zhou Y, Kijlstra A, Yang P (2014). Polymorphisms in genetics of vitamin D metabolism confer susceptibility to ocular Behçet disease in a Chinese Han population. Am J Ophthalmol.

[39] Uitterlinden AG, Fang Y, van Meurs JB, van Leeuwen H, Pols HA (2004). Vitamin D receptor gene polymorphisms in relation to Vitamin D related disease states. J Steroid Biochem Mol Biol.

[40] Pilz S, Tomaschitz A (2010). Role of vitamin D in arterial hypertension. Expert Rev Cardiovasc Ther.

[41] Wuerzner G, Burnier M, Waeber B (2012). Should hypertensive patients take vitamin D?. Curr Hypertens Rep.

[42] Swapna N, Vamsi UM, Usha G, Padma T (2011). Risk conferred by FokI polymorphism of vitamin D receptor (VDR) gene for essential hypertension. Indian J Hum Genet.

[43] Poon AH, Laprise C, Lemire M, Montpetit A, Sinnett D, Schurr E, Hudson TJ (2004). Association of vitamin D receptor genetic variants with susceptibility to asthma and atopy. Am J Respir Crit Care Med.

[44] Raby BA, Lazarus R, Silverman EK, Lake S, Lange C, Wjst M, Weiss ST (2004). Association of vitamin D receptor gene polymorphisms with childhood and adult asthma. Am J Respir Crit Care Med.

[45] Wjst M (2005). Variants in the vitamin D receptor gene and asthma. BMC Genet.

[46] Feng M, Li H, Chen SF, Li WF, Zhang FB (2012). Polymorphisms in the vitamin D receptor gene and risk of autoimmune thyroid diseases: a meta-analysis. Endocrine.

[47] Wang TJ, Zhang F, Richards JB, Kestenbaum B, van Meurs JB, Berry D, Kiel DP, Streeten EA, Ohlsson C, Koller DL, Peltonen L, Cooper JD, O’Reilly PF, Houston DK, Glazer NL, Vandenput L, Peacock M, Shi J, Florez JC, Todd JA, Dupuis J, Hyppönen E, Spector TD (2010). Common genetic determinants of vitamin D insufficiency: a genome-wide association study. Lancet.

[48] Porter FD (2002). Malformation syndromes due to inborn errors of cholesterol synthesis. J Clin Invest.

[49] Zhang Y, Wang X, Liu Y, Qu H, Qu S, Wang W, Ren L, The GC (2012). CYP2R1 and DHCR7 genes are associated with vitamin D levels in northeastern Han Chinese children. Swiss Med Wkly.

[50] Capriotti E, Fariselli P, Casadio R (2005). I-Mutant2.0: predicting stability changes upon mutation from the protein sequence or structure. Nucleic Acids Res.

[51] Pires DE, Ascher DB, Blundell TL (2014). DUET: a server for predicting effects of mutations on protein stability using an integrated computational approach. Nucleic Acids Res.

[52] Pires DE, Ascher DB, Blundell TL (2014). mCSM: predicting the effects of mutations in proteins using graph-based signatures. Bioinformatics.

[53] Worth CL, Preissner R, Blundell TL (2011). SDM--a server for predicting effects of mutations on protein stability and malfunction. Nucleic Acids Res.

